# Tuberous Sclerosis Complex–Associated Tubulointerstitial Kidney Disease

**DOI:** 10.1016/j.ekir.2025.04.001

**Published:** 2025-04-05

**Authors:** Hamza Sakhi, Pierre Isnard, Idris Boudhabhay, Jean-Michel Correas, Stephane Burtey, Dominique Joly, Bertrand Knebelmann, Aude Servais

**Affiliations:** 1 Department of Nephrology, Necker-Enfants Malades University Hospital, APHP, Université Paris Cité, Paris, France; 2 Department of Pathology, Necker-Enfants Malades University Hospital, APHP, Université Paris Cité, Paris, France; 3 Department of Adult Radiology, Necker-Enfants Malades University Hospital, APHP, Université Paris Cité, Paris, France; 4 Centre de Néphrologie et Transplantation Rénale, Hôpital de la Conception, Marseille, France; 5 Aix Marseille Université, INSERM 1263, INRAE 1260, C2VN, Marseille, France

**Keywords:** tuberous sclerosis complex, tubulointerstitial nephritis

## Introduction

Tuberous sclerosis complex (TSC) is a rare autosomal dominant genetic disease caused by *TSC1* or *TSC2* mutations, leading to mammalian target of rapamycin (mTOR) overactivation.^1^ It is characterized by various benign tumors. Renal involvement mainly includes angiomyolipomas (AMLs) or renal cysts. Chronic kidney disease (CKD) in patients with TSC is rare and is usually attributed to nephron reduction related to AML size, ablative therapy, or surgery.^2^, ^3^, ^4^ According to various cohorts, the prevalence of stage 2 CKD is 25% and that of stage 3 CKD approximately 7%, with a median age of onset of renal failure in the fourth decade of life.^4^^,^S1 Of particular interest, some patients with TSC develop CKD without large AML and/or invasive therapies. We performed a clinicopathologic evaluation of 7 patients with TSC with CKD, providing further insights into a potential new form of TSC-associated tubulointerstitial kidney disease.

## Results

### Clinical Presentation

Seven patients (6 females) with a sporadic TSC disease and a median age of 23 (18–60) years at kidney biopsy, were included. Genetic analysis, available for 6 patients revealed a mutation in *TSC1* and *TSC2* for 4 and 2 patients respectively with typical clinical TSC features for the last (Supplementary Tables S1 and S2). Renal involvement was characterized by near normal kidney aspect on imaging with only few intraparenchymal microcysts in all patients but 2 with limited number of small AMLs (Supplementary Figure S1) and no embolization, ablative, or surgical procedure. Median estimated glomerular filtration rate was 43 (28–88) ml/min per 1.73 m^2^. Median urine protein creatinine ratio was 1 (0.4–2.3) g/g (Supplementary Tables S1 and S3).

### Pathological Analysis

**Figure 1:**
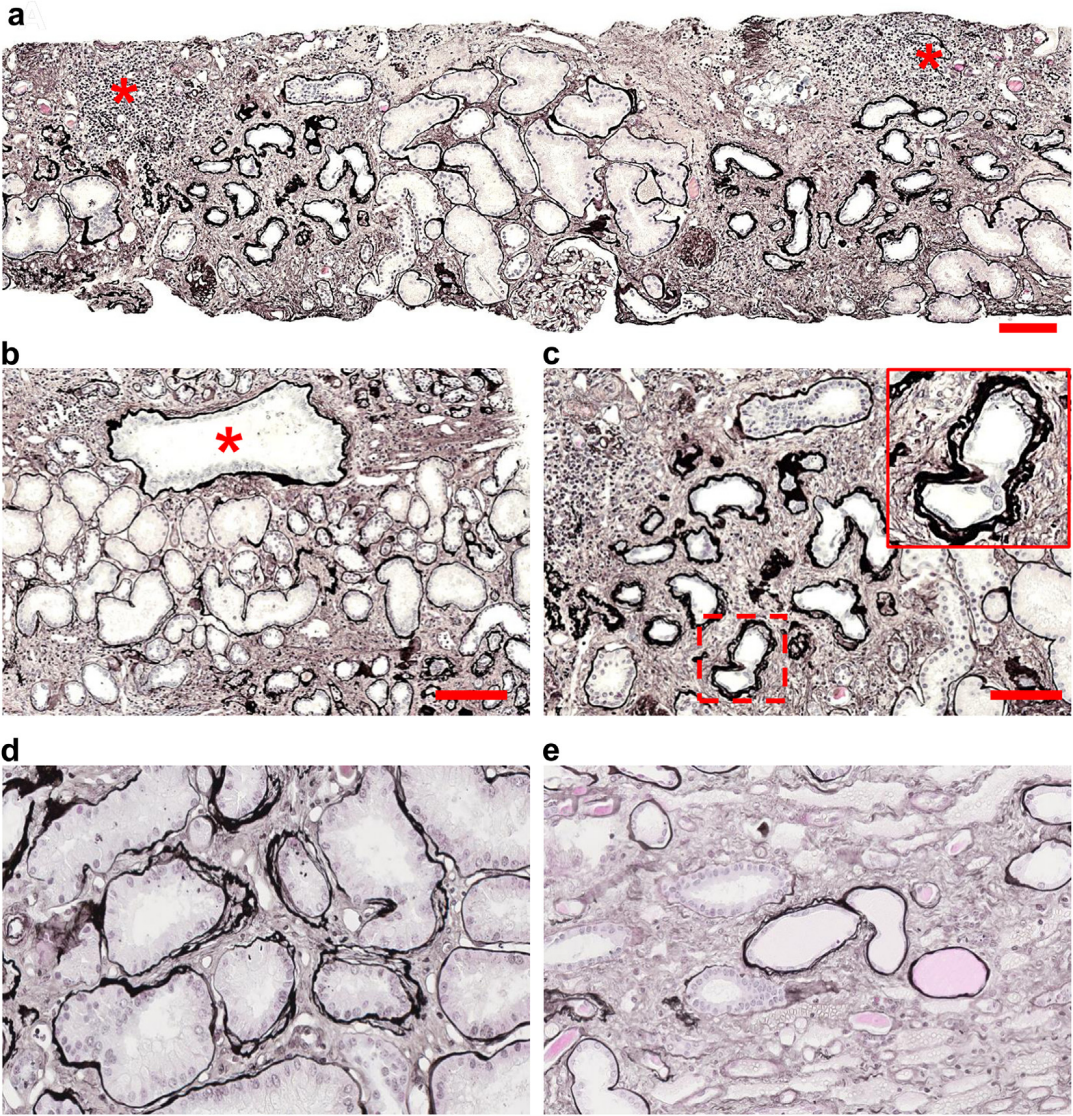
(a) Representative image of patient’s kidney biopsies using light microscopy with silver staining, mainly showing areas of interstitial fibrosis with tubular atrophy and areas of inflammatory fibrosis (red asterisk). (b) Light microscopy using silver staining showing cyst-like dilatation of some tubular sections (red asterisk). (c) Light microscopy using silver staining showing frequent lamellation and thickening of tubular basement membranes (red square). (d and e) Light microscopy using silver staining showing these nephronophthisis-like changes in less atrophic tubules (d) in the cortex and (e) in 1 medulla sample. Scale bar: 100 μm.

Kidney biopsies were performed in all 7 patients. The main pathological findings are illustrated in Figure 1 and summarized in Supplementary Table S4 and S5). All biopsies showed a similar pattern. The mean percentage of globally sclerosed glomeruli was 59% with 2 patients presenting with focal segmental glomerulosclerosis. No specific glomerular disease was identified, and immunofluorescence analyses were negative.

Interstitial fibrosis and tubular atrophy were present in all patients involving > 25% of the renal tissue. In 4 patients, interstitial fibrosis and tubular atrophy was extending to > 50% of the parenchyma and was associated with interstitial inflammation composed of lymphocytes and macrophages, covering 25% to 50% of these areas. No tubulitis lesions were observed. The remaining tubulointerstitial sectors showed mainly hypertrophic tubules, and 4 patients had pseudocystic tubules (Figure 1a and b). Tubular basement membranes appeared thickened, laminated, and wrinkled, as usually seen in nephronophthisis (Figure 1c–e).^5^ Overall, these findings suggest an atypical chronic tubulointerstitial nephritis (CTIN).

**Figure 2:**
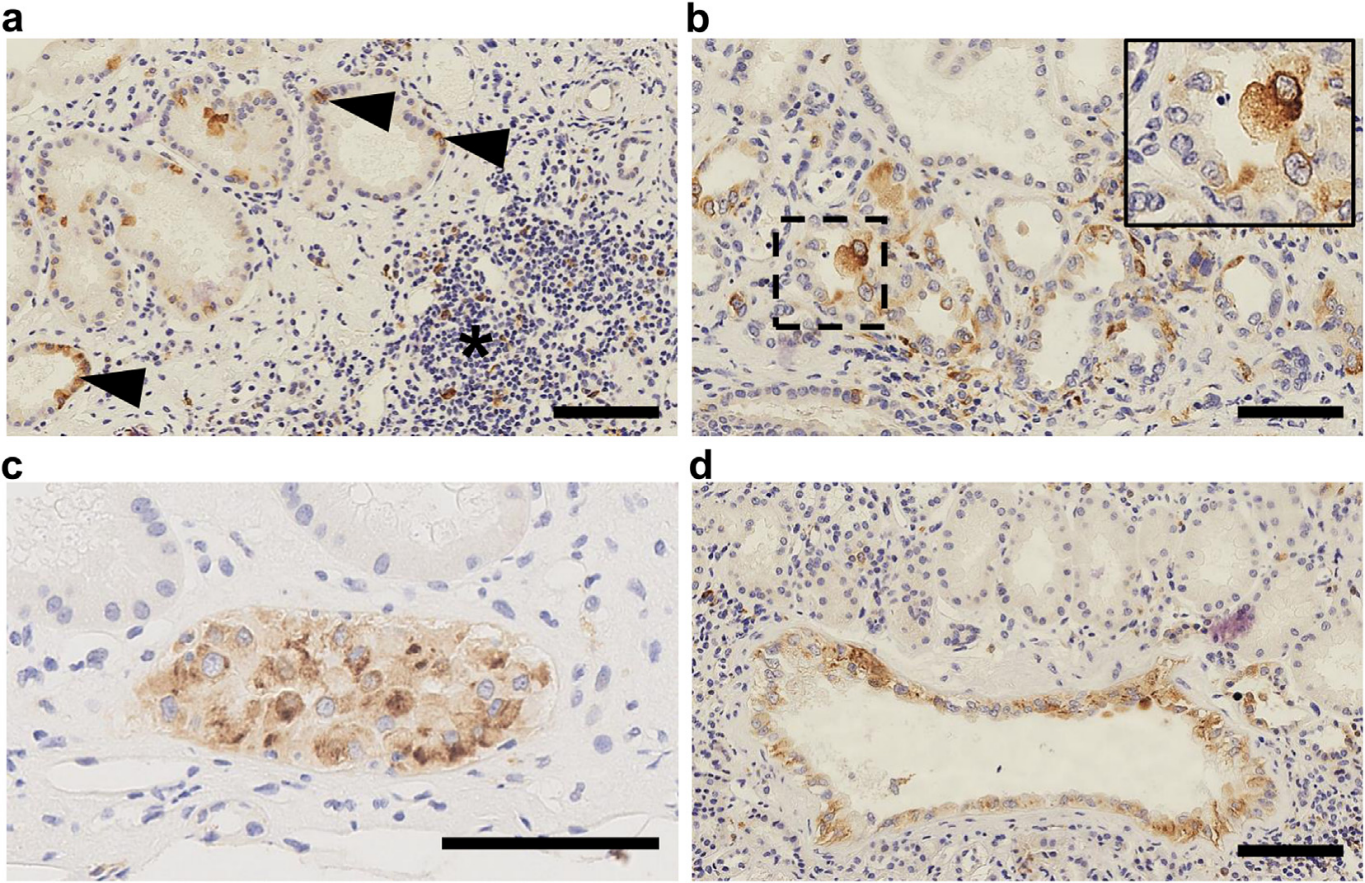
(a) Representative image of patient’s kidney biopsies with immunohistochemistry analysis targeting P-S6RP showing positivity of certain contiguous or scattered tubular epithelial cells (black arrow) and lymphocytes in fibro-inflammatory areas (black asterisk). (b) Immunohistochemistry analysis targeting P-S6RP showing positive tubular cells within dystrophic tubules and in cells with often hypertrophic nuclei (black square). (c) Immunohistochemistry analysis targeting P-S6RP showing a strongly positive cyst with several layers of tubular epithelial cells. (d) Immunohistochemistry analysis targeting P-S6RP showing a positive cyst-like dilatation of some tubular sections. Scale Bar: 100 μm. P-S6RP, phosphorylated S6 ribosomal protein.

To determine mTOR pathway activation in the kidney, we performed immunohistochemistry analysis targeting phosphorylated S6 ribosomal protein. We found labeling of tubular epithelial cells, and some lymphocytes within the interstitium (Figure 2a, c, and d**)**. We observed positivity of some contiguous or scattered tubular epithelial cells within dystrophic tubules and in cells with often hypertrophic nuclei (Figure 2b**)**. Pseudocystic tubules were particularly positive (Figure 2c and d). These results suggest that mTOR pathway activation was not limited to epithelial cystic or tumoral lesions in patients with TSC but may also be involved in the genesis of tubular and possibly interstitial lesions observed in our patients.

To determine mTOR activation specificity, phosphorylated S6 ribosomal protein staining was evaluated in normal kidneys (*n* = 4), acute tubular necrosis (ATN) (*n* = 4), acute interstitial nephritis (AIN) (*n* = 4), and CTIN (*n* = 4). Normal kidneys showed no staining (Supplementary Figure S2A). Acute tubular necrosis had low, heterogeneous positivity (Supplementary Figure S2B). Acute interstitial nephritis and CTIN showed strong positivity in infiltrating leukocytes, but dystrophic tubules lacked significant staining, in contrast to our TSC cases (Supplementary Figure S2C and D).

### Differential Diagnosis

Other potential causes of CTIN were excluded in these patients (especially exposure to drugs such as lithium or family history of other possible genetic disorders [nephronophthisis, autosomal dominant tubulointerstitial kidney disease, or autosomal dominant polycystic kidney disease]). Autosomal dominant tubulointerstitial kidney disease panel and autosomal dominant polycystic kidney disease mutation testing were performed in 1 patient each, with negative results.

### Follow-Up

Median follow-up was 69 (range 12–193) months, with a median age of 33 (range: 24–66) years at last follow-up. Patient 5 died 3 years after renal diagnosis at the age of 26 years because of severe cardiac dysfunction, potentially related to TSC.^S2^ Five of 7 patients exhibited rapid renal decline, progressing to CKD stage 4 to 5, with median estimated glomerular filtration rate of 17 (range: 9–58) ml/min per 1.73 m^2^ at last follow-up and a median annual decrease of −9 (range: 3.5–39.5) ml/min per 1.73 m^2^) (Supplementary Table S1 and Supplementary Figure S3).S5

Three patients received mTOR inhibitors as follows: patient 5 for 32 months, with estimated glomerular filtration rate declining from 86 to 58; patient 6 for 6 months, discontinued because of recurrent bronchitis; and patient 7 for AMLs with sirolimus, progressing to end-stage kidney disease 5 years later (Supplementary Table S3).

## Discussion

TSC-associated CKD typically occurs in patients with large AMLs and/or a history of renal intervention leading to nephron reduction.^2^, ^3^, ^4^ In this study, we performed a detailed clinicopathologic evaluation of TSC-associated CKD with atypical chronic tubulointerstitial lesions in patients without large kidney lesions or previous renal interventions, providing further insights into a recently reported interstitial nephropathy.^6^ Kronick *et al.*^6^ described this CKD as characterized by early renal dysfunction (median onset: 23 years) and rapid estimated glomerular filtration rate decline, with 2 patients reaching end-stage renal disease ages at 23 and 27 years.^6^

Pathologically, Kronick *et al.*^6^ observed severe interstitial fibrosis, tubular atrophy, and irregular tubular dilatations. In our study of 7 patients, the dominant pattern was nephronophthisis-like picture, with tubular dilatations and focal laminated aspects of the tubular basement membrane.^5^ We also found significant interstitial inflammation.

Regarding pathophysiology, TSC inactivation and mTOR activation in renal tubular epithelial cells are associated with tubular dedifferentiation, interstitial fibrosis, and cyst formation.^7^, ^8^, ^9^ As in nephronophthisis, inflammatory mediators secreted by tubular cells can trigger the recruitment of immune cells in response to mTOR activation.S3 We observed a strong expression of phosphorylated S6 ribosomal protein in dystrophic tubular epithelial cells, suggesting an overactivation of the mTOR pathway in this tubular cells. Whether this relates to a somatic second hit or to *TSC1/TSC2* haploinsufficiency remains to be determined.

mTOR activation was also present, albeit lower and more irregular, in tubular cells from patients with acute tubular necrosis, possibly reflecting epithelial regeneration. However, the frequence of dystrophic tubules and their consistent phosphorylated S6 ribosomal protein staining suggests some specificity associated with TSC-related CTIN. Moreover, mTOR overexpressing tubular cells could potentially contribute to renal fibrosis.S4 During CKD, activation of mTOR signaling in nonepithelial cells such as fibroblasts and macrophages promotes renal fibrosis and may contribute to our newly described TSC-associated kidney disease.S4

Activation of the mTOR pathway in tubular cells questions the potential value of using mTOR inhibition. However, though mTOR inhibitors have shown efficacy in other TSC-related conditions,S2 further studies are needed to determine whether mTOR inhibition has a beneficial effect in TSC-associated CKD.^6^

In summary, we report a detailed clinicopathologic study on TSC-associated CTIN. Whether this specific pattern represents a rare and atypical form of TSC kidney involvement or a mechanism contributing to CKD in all patients with TSC remains uncertain because of the rarity of kidney biopsies. A multicenter cohort study could hopefully answer this puzzling question.

## Disclosure

All the authors declared no competing interests.

## Author Contributions

HS, PI, AS, and BK designed the study. HS and PI collected data. IB, DJ, JMC, and SB cared for the study patients. HS, PI, BK, and AS analyzed the data. HS, PI, BK, and AS wrote the paper. All the authors provided feedback, critical review, and approved the final version of the manuscript.

## Data Availability Statement

All data presented in this study are included in the main manuscript and Supplementary Tables. Additional underlying data can be obtained from the corresponding author upon reasonable request.

## Supplementary Material

Supplementary File (PDF) **Supplementary Methods. Supplementary References. Figure S1.** Magnetic resonance images of tuberous sclerosis complex kidney showing microcystic aspect with no angiomyolipoma observed. **Figure S2.** Representative images of patient’s kidney biopsies with immunohistochemistry analysis targeting phosphorylated S6 ribosomal protein. **Figure S3.** Spaghetti plots of estimated glomerular filtration rate through follow up. **Table S1.** Tuberous sclerosis complex features and renal characteristics at diagnosis. **Table S2.** Tuberous sclerosis complex features for each patient. **Table S3.** Renal characteristics at diagnosis and at last follow-up for each patient. **Table S4.** Histological parameters of patients with tuberous sclerosis complex. **Table S5.** Histological parameters for each patient with tuberous sclerosis complex. STROBE Checklist.

## Footnotes

Supplementary File (PDF)

**Supplementary Methods.**

**Supplementary Re** **ferences.**
**Figure S1.** Magnetic resonance images of tuberous sclerosis complex kidney showing microcystic aspect with no angiomyolipoma observed. **Figure S2.** Representative images of patient’s kidney biopsies with immunohistochemistry analysis targeting phosphorylated S6 ribosomal protein. **Figure S3.** Spaghetti plots of estimated glomerular filtration rate through follow up. **Table S1.** Tuberous sclerosis complex features and renal characteristics at diagnosis. **Table S2.** Tuberous sclerosis complex features for each patient. **Table S3.** Renal characteristics at diagnosis and at last follow-up for each patient. **Table S4.** Histological parameters of patients with tuberous sclerosis complex. **Table S5.** Histological parameters for each patient with tuberous sclerosis complex. STROBE Checklist.
