# Supplementary material for: Tuberous Sclerosis Complex–Associated Tubulointerstitial Kidney Disease
Source: Kidney Int Rep. 2025 Apr 5;10(6):2049–53. doi: 10.1016/j.ekir.2025.04.001 (PMC12231016; doi:10.1016/j.ekir.2025.04.001)
Supplement: Supplementary File (PDF) — Supplementary Methods. Supplementary References. Figure S1. Magnetic resonance images of tuberous sclerosis complex kidney showing microcystic aspect with no angiomyolipoma observed. Figure S2. Representative images of patient’s kidney biopsies with immunohistochemistry analysis targeting phosphorylated S6 ribosomal protein. Figure S3. Spaghetti plots of estimated glomerular filtration rate through follow up. Table S1. Tuberous sclerosis complex features and renal characteristics at diagnosis. Table S2. Tuberous sclerosis complex features for each patient. Table S3. Renal characteristics at diagnosis and at last follow-up for each patient. Table S4. Histological parameters of patients with tuberous sclerosis complex. Table S5. Histological parameters for each patient with tuberous sclerosis complex. STROBE Checklist. [file mmc1.pdf]

Supplementary materials

Supplementary methods

Supplementary table S1. Tuberous sclerosis complex features and renal characteristics at diagnosis

Supplementary table S2. Tuberous sclerosis complex features for each patient

Supplementary table S3. Renal characteristics at diagnosis and last follow up for each patient

Supplementary table S4. Histological parameters of TSC patients

Supplementary table S5. Histological parameters for each TSC patient

Supplementary figure S1. Magnetic resonance images of TSC kidney showing microcystic aspect with no angiomyolipoma observed.

Supplementary figure S2. Representative images of patient's kidney biopsies with immunohistochemistry analysis targeting P-S6RP

Supplementary figure S3. Spaghetti plots of estimated glomerular filtration rate through follow up.

Supplementary References

Strobe Statement

Supplementary methods

Tuberous Sclerosis Complex (TSC) patients who underwent kidney biopsy due to kidney dysfunction and/or proteinuria from two French centers (Hôpital Necker, Paris and Hôpital de la Conception, Marseille) between December 2005 and April 2022 were included in this study (n=7). Clinical, biological, and imaging data were retrospectively retrieved from medical records. Demographic characteristics included sex and age at the time of TSC diagnosis and kidney biopsy. Clinical data included past medical history, specifically hypertension (either prior to or at the time of diagnosis), and conditions associated with TSC, such as identified gene variants (when available), neurological, dermatological, pulmonary, and kidney manifestations. Biological data included serum creatinine levels with estimated glomerular filtration rate (eGFR) calculated using the CKD-EPI formula (S5) at the time of kidney biopsy, as well as urine protein-to-creatinine ratio measurements. Radiological findings were based on abdominal magnetic resonance images or computed tomography scans.

Pathological data were extracted from pathology reports, and six out of seven biopsies were blindly re-evaluated by a kidney pathologist. Glomerular, tubulointerstitial, and vascular lesions, as well as immunofluorescence deposits, were assessed. The degree of lesions in each compartment was categorized using a semi-quantitative scoring system: absence (0-10%), mild (10-25%, 1), moderate (25-50%, 2), and severe (≥50%, 3). For six out of seven patients, automated immunohistochemistry (IHC) analysis of P-S6RP was performed using the Leica BOND-III (Leica Biosystems) module and the anti-P-S6RP (Ser240/244, D68F8) antibody.

At the last follow-up, data collected included serum creatinine levels with eGFR, onset of end-stage renal disease, kidney transplantation, and death.

Continuous variables were expressed as medians with minimum and maximum values, while categorical variables were expressed as absolute numbers with percentages.

Supplementary table S1. Tuberous sclerosis complex features and renal characteristics at diagnosis

VARIABLES	Patients n = 7
**Epidemiological data**	
Sex (female), n (%)	6 (85)
**Tuberous sclerosis complex features**	
Age at diagnosis (years), median (min-max)	15 (1-26)
gene n (%)	
TSC1	4/6 (67)
TSC2	2/6 (33)
Neurological features	
Cortical tubers, n (%)	5 (71)
Epilepsy, n (%)	3 (43)
Cognitive deficit, n (%)	3 (43)
Brain tumor, n (%)	1 (14)
Pulmonary features	
Lymphangioleiomyomatosis, n (%)	1 (14)
Multifocal micronodular pneumocyte hyperplasia, n (%)	1 (14)
Dermatological features	
Hypopigmented macules, n (%)	3 (43)
Angiofibroma, n (%)	4 (57)
Ungual fibroma, n (%)	1 (14)
Kidney features	
Angiomyolipoma, n (%)	2 (29)
Cystic kidneys, n (%)	7 (100)
**Kidney presentation**	
Age at kidney biopsy (years), median (min-max)	23 (18-60)
Serum creatinine (µmol/l), median (min-max)	140 (83-190)
eGFR (ml/min/1.73m2), median (min-max)	43 (28-88)
Urine protein creatinine ratio (g/g), n (%)	1.1 (0.4-2.3)
Hypertension, n (%)	4 (57)
**Follow-up**	
Time of follow up (months), median (min-max)	69 (12-193)
Serum creatinine (µmol/l), median (min-max)	232 (90-464)
eGFR (ml/min/1.73m2), median (min-max)	17 (9-58)
eGFR < 15 ml/min/1.73m2, n (%)	3 (43)
Death, n (%)	1 (14)

Supplementary table S2. Tuberous sclerosis complex features for each patient

**Pat.**	**Sex**	**TSC features**							
		**Age at TSC diagnosis**	**Gene**	**Neuro.**	**Heart**	**Pulm**	**Cutaneous**	**Renal AML**	**Renal microcysts** ~~**M.Cyst**~~
**P1**	M	1	*TSC1*	C.T/Ep/C.D	No	No	Ungual fibroma / Hypopigmented macules	No	Yes
**P2**	F	4	*TSC2*	Ep/ Brain tumor	No	No	No	No	Yes
**P3**	F	26	*TSC1*	C.T / Ep/ C.D	No	No	Angiofibroma	No	Yes
**P5**	F	15	*TSC1*	C.T / Ep/ C.D	No	No	Hypopigmented macules / Angiofibroma	No	Yes
**P5**	F	10	*TSC 2*	C.D	No	No	Angiofibroma	One	Yes
**P6**	F	23	*TSC1*	C.T	No	MMPH	Hypopigmented macules / Angiofibroma	No	Yes
**P7**	F	20	-	-	No	Llm	-	Yes	Yes

AML: Angiomyolipoma; C.T : Cortical tubers ; Ep : Epilepsy ; C.D : Cognitive deficit; Pulm : pulmonary ; TSC: Tuberous Sclerosis Complex ; MMPH : Multifocal micronodular pneumocyte hyperplasia ; Llm : Lymphangioleiomyomatosis

Supplementary table S3. Renal characteristics at diagnosis for each patient

Pat.	Kidney parameters at diagnosis					Last follow-up						
	**Age (years)**	**Creat (µmol/l)**	**eGFR (ml/min/1.73m2)**	**Proteinuria (g/g)**	**HT**	**Time (mt)**	**Creat (µmol/l)**	**Age at last Fup (years)**	**eGFR (ml/min)**	**ESRD**	**Age at ESRD (years)**	**Other**
**P1**	18	106	88	0.78	Yes	193	90 (RT)	34	96	Yes	22	Renal transplantation
**P2**	23	140	46	2	No	120	464	33	10	Yes	27	
**P3**	31	140	43	0.4	Yes	78	293	37	17	No	-	Lymphe node & hypercalcemia Sarcoidosis onset during Fup
**P4**	23	190	32	1.1	No	13	232	24	25	No	-	
**P5**	23	83	86	1.29	No	32	118	26	58	No	-	Death from cardiac dysfunction
**P6**	22	146	43	1	Yes	12	163	24	38	No	-	
**P7**	60	162	28	2.28	Yes	69	406	66	9	Yes	65	

Creat. : Creatinine ; eGFR : estimated glomerular filtration rate ; ESRD : End-stage renal disease ; Fup : Follow-up ; Ht ; Hypertension ; mt : months; Pat : Patient

Supplementary table S4. Pathological parameters of TSC patients

**VARIABLES**	**Patients<br>N = 7**
**Glomerular lesions**	
Total number of glomeruli, mean (SD)	11 (± 6)
Globally sclerotic glomeruli, mean (SD)	5 (± 2)
Ratio of sclerotic glomeruli (%), mean (SD)	59 (± 20)
Focal segmental glomerulosclerosis, n (%)	2 (29)
**Tubular and interstitial lesions**	
Interstitial fibrosis and Tubular atrophy (0-3+), mean (SD)	2 (± 0.7)
Interstitial inflammation (0-3+), mean (SD)	0.15 (± 0.4)
Interstitial inflammation within fibrosis (0-3+), mean (SD)	2 (± 0.7)
Tubulitis, n (%)	0 (0)
Cystic tubules, n (%)	4 (57)
Lamellation of tubular basement membranes, n (%)	6 (86)
Thickening of tubular basement membranes, n (%)	6 (86)
**Vascular lesions**	
Arteriosclerosis (0-3+), mean (SD)	1 (± 0.6)
Arteriolar hyalinosis (0-3+), mean (SD)	1 (± 0.7)
**Immunoflurorescence (deposits), n (%)**	0 (0)

Supplementary table S5. Pathological parameters for each TSC patient

Pat	Date of Kidney Biopsy	Glomerular lesions				Tubular and interstitial lesions							Vascular lesions		IF
		Total number of glomeruli	Globally sclerotic glomeruli	Ratio of sclerotic glomeruli	Glomerular lesion	Interstitial fibrosis and Tubular atrophy (0-3+)	Interstitial inflammation (0-3+)	Interstitial inflammation within fibrosis (0-3+)	Tubulitis (Y-N)	Cystic Tubules (Y-N)	Lamellation of tubular basement membranes	Thickening of tubular basement membranes	Arteriosclerosis (0-3+)	Arteriolar hyalinosis (0-3+)	
P1	22/12/05	6	4	67%	No	2	0	2	No	Yes	Yes	Yes	1	1	(-)
P2	27/11/12	5	4	80%	No	3	0	3	No	Yes	Yes	Yes	0	0	(-)
P3	07/03/13	11	5	45%	No	2	1	2	No	Yes	Yes	Yes	1	1	(-)
P4	01/04/21	20	8	40%	No	3	0	2	No	Yes	Yes	Yes	1	2	(-)
P5	05/02/15	6	3	50%	NOS FSGS	2	0	1	No	No	Yes	Yes	1	1	(-)
P6	07/04/22	17	7	41%	NOS FSGS	3	0	2	No	No	Yes	Yes	1	1	(-)
P7	03/08/18	9	8	89%	No	3	0	3	No	No	No	No	2	2	(-)

Pat : patients ; IF : immunofluorescence

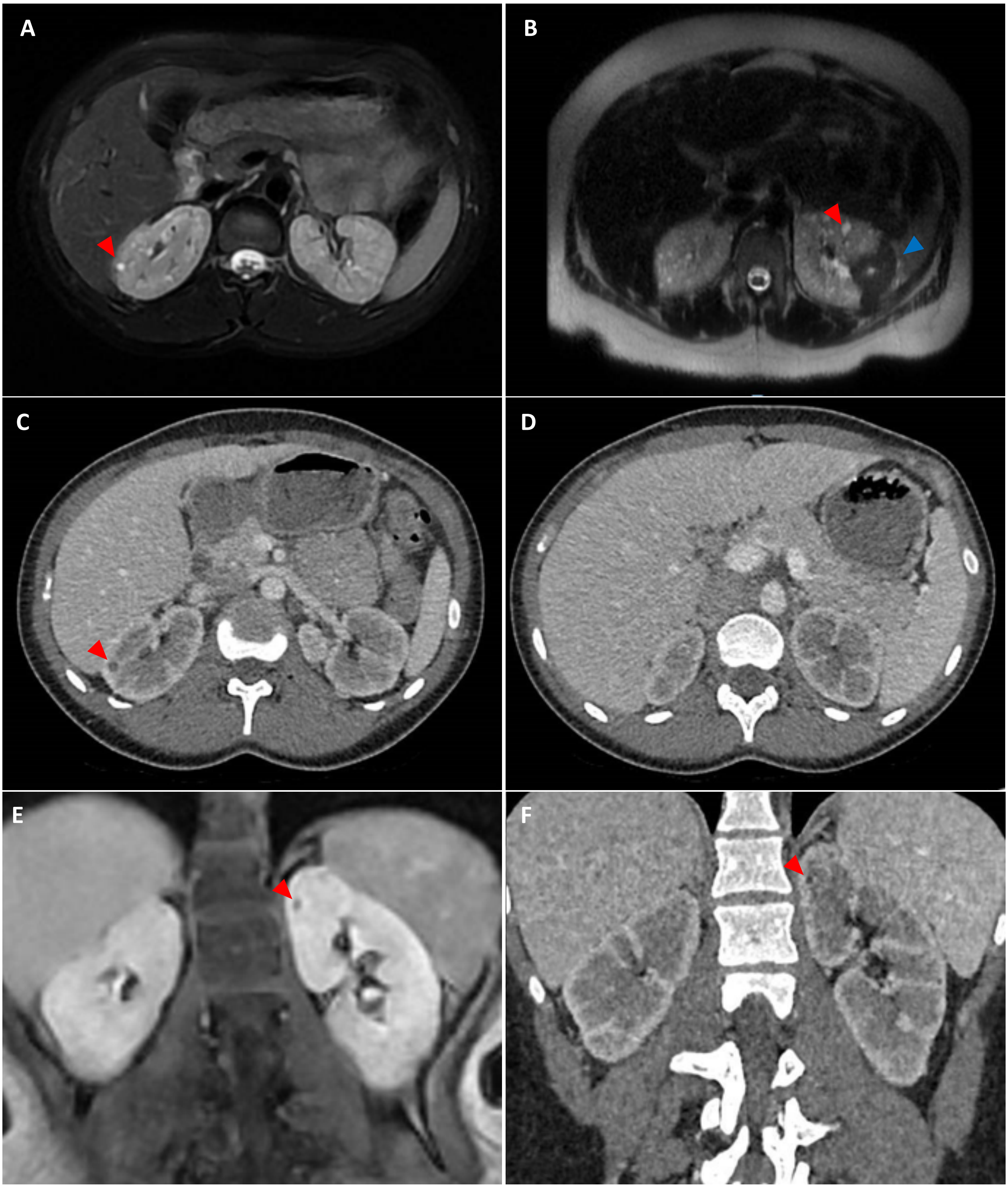

Supplementary figure S1. Magnetic resonance images (MRI) and Computed tomography CT-scan of TSC kidney showing near normal kidneys with few microcystic (red arrow) and with no angiomyolipoma observed (Patient 6) or isolated angiomyolipoma (blue arrow, Patient 5) . (**A**) and (**B**) Axial T2-weighted MR images; (**C**) and (**D**) Axial contrast-enhanced CT image; (**E**) coronal lavaflex MR image and (**F**) coronal Axial contrast-enhanced CT image

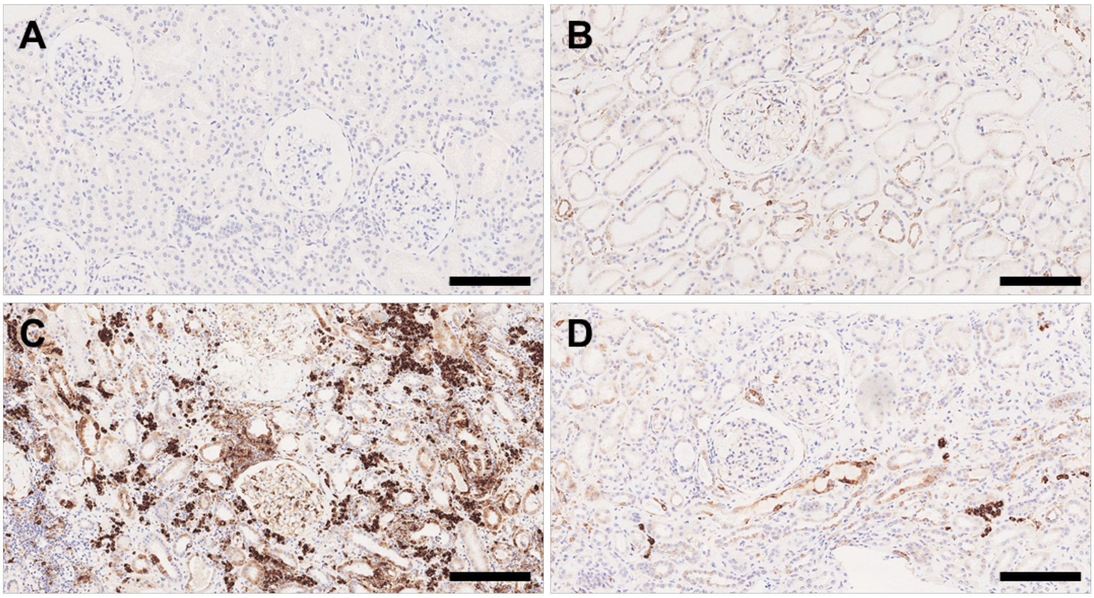

Supplementary figure S2. **Representative images of patient's kidney biopsies with immunohistochemistry analysis targeting P-S6RP** in : **A.** morphologically normal kidneys. **B.** acute tubular necrosis (ATN) lesions, **C.** acute interstitial nephritis (AIN) and **D.** chronic tubule-interstitial nephritis (CTIN). Scale Bare 100µm.

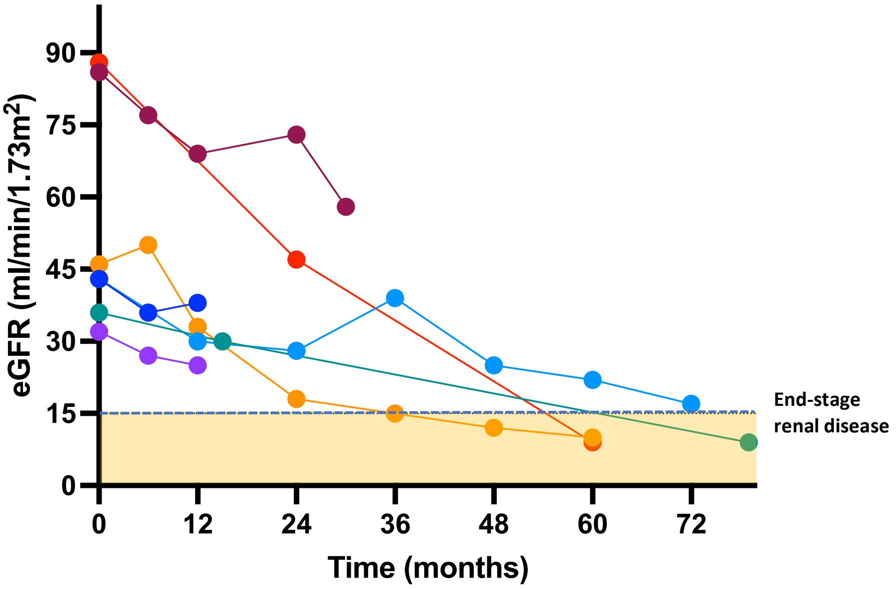

Supplementary figure S3. Spaghetti plot of estimated glomerular filtration rate (ml/min/1.73m^2) through follow up (months). Patient 1 : red ; Patient 2 : yellow ; Patient 3 : light blue ; Patient 4 : violet ; Patient 5 : purple ; Patient 6 : dark blue ; Patient 7 : green.

Supplementary References

S1. Janssens P, Van Hoeve K, De Waele L, De Rechter S, Claes KJ, Van de Perre E, et al. Renal progression factors in young patients with tuberous sclerosis complex: a retrospective cohort study. Pediatr Nephrol. nov 2018;33(11):2085-93.

S2. Koyama M, Yano T, Kikuchi K, Miura T. Everolimus-responsive dilated cardiomyopathy in tuberous sclerosis. European Heart Journal. 7 sept 2015;36(34):2338.

S3. Quatredeniers M, Bienaimé F, Ferri G, Isnard P, Porée E, Billot K, et al. The renal inflammatory network of nephronophthisis. Hum Mol Genet. 7 juill 2022;31(13):2121-36.

S4. Gui Y, Dai C. mTOR Signaling in Kidney Diseases. Kidney360. 3 sept 2020;1(11):1319-27

S5. Levey AS, Stevens LA, Schmid CH, Zhang YL, Castro AF, Feldman HI, et al. A new equation to estimate glomerular filtration rate. Ann Intern Med. 5 mai 2009;150(9):604-12

STROBE Statement—checklist of items that should be included in reports of observational studies

	Item No.	Recommendation	Page No.	Relevant text from manuscript
**Title and abstract**	1	(*a*) Indicate the study's design with a commonly used term in the title or the abstract	Title, p1	
		(*b*) Provide in the abstract an informative and balanced summary of what was done and what was found	n.a	
**Introduction**				
Background/rationale	2	Explain the scientific background and rationale for the investigation being reported	p2	
Objectives	3	State specific objectives, including any prespecified hypotheses	p2	
**Methods**				
Study design	4	Present key elements of study design early in the paper	supp.material p2-supp methods	
Setting	5	Describe the setting, locations, and relevant dates, including periods of recruitment, exposure, follow-up, and data collection	supp.material p2-supp methods	
Participants	6	(*a*) *Cohort study*—Give the eligibility criteria, and the sources and methods of selection of participants. Describe methods of follow-up<br>*Case-control study*—Give the eligibility criteria, and the sources and methods of case ascertainment and control selection. Give the rationale for the choice of cases and controls<br>*Cross-sectional study*—Give the eligibility criteria, and the sources and methods of selection of participants	supp.material p2-supp methods	
		(*b*) *Cohort study*—For matched studies, give matching criteria and number of exposed and unexposed<br>*Case-control study*—For matched studies, give matching criteria and the number of controls per case	n.a	
Variables	7	Clearly define all outcomes, exposures, predictors, potential confounders, and effect modifiers. Give diagnostic criteria, if applicable	supp.material p2-supp methods	
Data sources/ measurement	8*	For each variable of interest, give sources of data and details of methods of assessment (measurement). Describe comparability of assessment methods if there is more than one group	supp.material p2-supp methods	
Bias	9	Describe any efforts to address potential sources of bias	supp.material	

			p2-supp methods
Study size	10	Explain how the study size was arrived at	supp.material p2-supp methods

Continued on next page

Quantitative variables	11	Explain how quantitative variables were handled in the analyses. If applicable, describe which groupings were chosen and why	Supp materials p2
Statistical methods	12	(*a*) Describe all statistical methods, including those used to control for confounding	n.a
		(*b*) Describe any methods used to examine subgroups and interactions	n.a
		(*c*) Explain how missing data were addressed	n.a
		(*d*) *Cohort study*—If applicable, explain how loss to follow-up was addressed<br>*Case-control study*—If applicable, explain how matching of cases and controls was addressed<br>*Cross-sectional study*—If applicable, describe analytical methods taking account of sampling strategy	n.a
		(*e*) Describe any sensitivity analyses	n.a
**Results**			
Participants	13*	(a) Report numbers of individuals at each stage of study—eg numbers potentially eligible, examined for eligibility, confirmed eligible, included in the study, completing follow-up, and analysed	n.a
		(b) Give reasons for non-participation at each stage	n.a
		(c) Consider use of a flow diagram	n.a
Descriptive data	14*	(a) Give characteristics of study participants (eg demographic, clinical, social) and information on exposures and potential confounders	p3<br>Supp table 1,2 & 3
		(b) Indicate number of participants with missing data for each variable of interest	Supp table 2,3 & 5
		(c) *Cohort study*—Summarise follow-up time (eg, average and total amount)	p5, Supp table 1
Outcome data	15*	*Cohort study*—Report numbers of outcome events or summary measures over time	p5<br>Supp table 1,3 & supp figure 2
		Case-control study—Report numbers in each exposure category, or summary measures of exposure	n.a
		Cross-sectional study—Report numbers of outcome events or summary measures	n.a
Main results	16	(*a*) Give unadjusted estimates and, if applicable, confounder-adjusted estimates and their precision (eg, 95% confidence interval). Make clear which confounders were adjusted for and why they were included	n.a
		(*b*) Report category boundaries when continuous variables were categorized	n.a

	(*c*) If relevant, consider translating estimates of relative risk into absolute risk for a meaningful time period	n.a

Continued on next page

Other analyses	17	Report other analyses done—eg analyses of subgroups and interactions, and sensitivity analyses	n.a
**Discussion**			
Key results	18	Summarise key results with reference to study objectives	p6
Limitations	19	Discuss limitations of the study, taking into account sources of potential bias or imprecision. Discuss both direction and magnitude of any potential bias	p6-7
Interpretation	20	Give a cautious overall interpretation of results considering objectives, limitations, multiplicity of analyses, results from similar studies, and other relevant evidence	p6-7
Generalisability	21	Discuss the generalisability (external validity) of the study results	p6-7
**Other information**			
Funding	22	Give the source of funding and the role of the funders for the present study and, if applicable, for the original study on which the present article is based	n.a

*Give information separately for cases and controls in case-control studies and, if applicable, for exposed and unexposed groups in cohort and cross-sectional studies.

Note: An Explanation and Elaboration article discusses each checklist item and gives methodological background and published examples of transparent reporting. The STROBE checklist is best used in conjunction with this article (freely available on the Web sites of PLoS Medicine at http://www.plosmedicine.org/, Annals of Internal Medicine at http://www.annals.org/, and Epidemiology at http://www.epidem.com/). Information on the STROBE Initiative is available at www.strobe-statement.org.
